# Revised Hammersmith Scale for spinal muscular atrophy: A SMA specific clinical outcome assessment tool

**DOI:** 10.1371/journal.pone.0172346

**Published:** 2017-02-21

**Authors:** Danielle Ramsey, Mariacristina Scoto, Anna Mayhew, Marion Main, Elena S. Mazzone, Jacqueline Montes, Roberto de Sanctis, Sally Dunaway Young, Rachel Salazar, Allan M. Glanzman, Amy Pasternak, Janet Quigley, Elizabeth Mirek, Tina Duong, Richard Gee, Matthew Civitello, Gihan Tennekoon, Marika Pane, Maria Carmela Pera, Kate Bushby, John Day, Basil T. Darras, Darryl De Vivo, Richard Finkel, Eugenio Mercuri, Francesco Muntoni

**Affiliations:** 1 Dubowitz Neuromuscular Centre, UCL Great Ormond Street Institute of Child Health, London, United Kingdom; 2 John Walton Muscular Dystrophy Research Centre, Institute of Genetic Medicine, Newcastle University, Newcastle, United Kingdom; 3 Department of Child Neurology, Catholic University in Rome, Rome, Italy; 4 Department of Neurology, Columbia University Medical Center, New York, New York, United States of America; 5 Department of Physical Therapy, The Children’s Hospital of Philadelphia, Philadelphia, Pennsylvania, United States of America; 6 Departments of Neurology and Physical Therapy and Occupational Therapy Services, Boston Children’s Hospital, Boston, Massachusetts, United States of America; 7 Department of Neurology, Stanford University, Palo Alto, California, United States of America; 8 Lucille Packard Children’s Hospital, Stanford University, Palo Alto, California, United States of America; 9 Nemours Children’s Hospital, University of Central Florida College of Medicine, Orlando, Florida, United States of America; 10 Department of Neurology, The Children’s Hospital of Philadelphia and the Pearlman School of Medicine, The University of Pennsylvania, Philadelphia, Pennsylvania, United States of America; Iowa State University, UNITED STATES

## Abstract

Recent translational research developments in Spinal Muscular Atrophy (SMA), outcome measure design and demands from regulatory authorities require that clinical outcome assessments are ‘fit for purpose’. An international collaboration (SMA REACH UK, Italian SMA Network and PNCRN USA) undertook an iterative process to address discontinuity in the recorded performance of the Hammersmith Functional Motor Scale Expanded and developed a revised functional scale using Rasch analysis, traditional psychometric techniques and the application of clinical sensibility via expert panels. Specifically, we intended to develop a psychometrically and clinically robust functional clinician rated outcome measure to assess physical abilities in weak SMA type 2 through to strong ambulant SMA type 3 patients. The final scale, the Revised Hammersmith Scale (RHS) for SMA, consisting of 36 items and two timed tests, was piloted in 138 patients with type 2 and 3 SMA in an observational cross-sectional multi-centre study across the three national networks. Rasch analysis demonstrated very good fit of all 36 items to the construct of motor performance, good reliability with a high Person Separation Index PSI 0.98, logical and hierarchical scoring in 27/36 items and excellent targeting with minimal ceiling. The RHS differentiated between clinically different groups: SMA type, World Health Organisation (WHO) categories, ambulatory status, and SMA type combined with ambulatory status (all *p* < 0.001). Construct and concurrent validity was also confirmed with a strong significant positive correlation with the WHO motor milestones *r*_*s*_ = 0.860, *p* < 0.001. We conclude that the RHS is a psychometrically sound and versatile clinical outcome assessment to test the broad range of physical abilities of patients with type 2 and 3 SMA. Further longitudinal testing of the scale with regards change in scores over 6 and 12 months are required prior to its adoption in clinical trials.

## Introduction

Spinal Muscular Atrophy (SMA) is the most common disease of the spinal motor neuron occurring in 1 in 6–10,000 births with a carrier frequency of 1 in 35–70 [1–5]. SMA is an autosomal recessive condition due in most cases to the homozygous deletion of the *SMN1* gene [2, 4–7]. There are four types of *SMN1*-related SMA, with types 1, 2 and 3 manifesting during infancy/childhood, while the type 4 onset is in adulthood [7]. Classification of SMA type depends upon the age of onset and highest level of motor function achieved [5–7]. Our study is focused on the SMA type 2 and 3 phenotype where the highest level of functional ability achieved is independent sitting in SMA 2, and standing or walking in SMA 3 [7].

With greater understanding of the molecular genetics of SMA over the past two decades, therapeutic interventions for this condition are rapidly entering phase 2 and 3 clinical trials [4, 8]. Evidence of efficacy of such therapeutics involves thorough assessment of the participant’s physical abilities through the use of functional scales [1, 7, 8]. Functional scales have been cited as important and recommended as primary outcome measures in clinical trials for the detection of meaningful changes and inclusion/exclusion criteria [9].

There has been much activity in the last fourteen years to develop a clinical outcome assessment to assess gross motor function in SMA types 2 and 3. The first SMA specific outcome measure, the Hammersmith Functional Motor Scale (HFMS), was developed in 2003 as both a clinical and research tool [10]. The HFMS is an assessment of the physical abilities of SMA type 2 and type 3 patients with limited ambulation. It is an ordinal scale consisting of twenty items with individual item scoring as 2 for unaided, 1 for performed with modification or adaption and 0 for unable [10]. The HFMS was widely adopted by the SMA community, however some revisions were implemented by several groups to improve its measurement capabilities. To remove any confounding effect of fatigue and effect of positional changes, the order of the HFMS was modified (MHFMS) [11, 12]. To enable its use in the ambulant type 3 population the HFMS was expanded to include 13 items from the Gross Motor Function Measure to form the Hammersmith Functional Motor Scale Expanded (HFMSE) [13]. Although not formally reported in the literature, the MHFMS was extended to include 8 additional gross motor items and timed tests resulting in the Modified Hammersmith Functional Motor Scale Extended (MHFMS-EXTEND) [14].

As an SMA specific outcome measure, the HFMSE is widely used internationally in clinical practice, in clinical trials and to document SMA natural history and trajectories of disease course [15–18]. The reliability, validity and sensitivity of change of the HFMSE was discussed at a recent international workshop on SMA outcome measures and the scale was found to fulfil the majority of criteria required by regulatory authorities [9]. Furthermore, the HFMSE is correlated with other aspects of SMA disease severity with positive associations found with *SMN2* copy number, compound muscle action potential (CMAP), forced vital capacity (FVC) and muscle strength, making it clearly a disease-specific outcome measure of choice for clinical trials [3, 19, 20].

While the HFMSE captures clinically relevant aspects of disease progression, some limitations with regards psychometric properties have been suggested [21, 22]. Rasch analysis has identified some discontinuities in its measurement properties and highlighted some issues with validity regarding measuring motor performance in different SMA phenotypes [21]. This background gave us the impetus to better define and assess the feasibility to further improve the well-established HFMSE and expand its utility and strength in both the clinical and scientific setting. Using the HFMSE as a foundation, this study aimed to develop a psychometrically and clinically robust functional clinician rated outcome measure to assess the spectrum of physical abilities from weak non-ambulant to strong ambulant patients with SMA types 2 and 3.

## Methods

### Construction & development of the Revised Hammersmith Scale for SMA (RHS)

#### Intent of scale—Concept of interest and context of use

**Fig 1:**
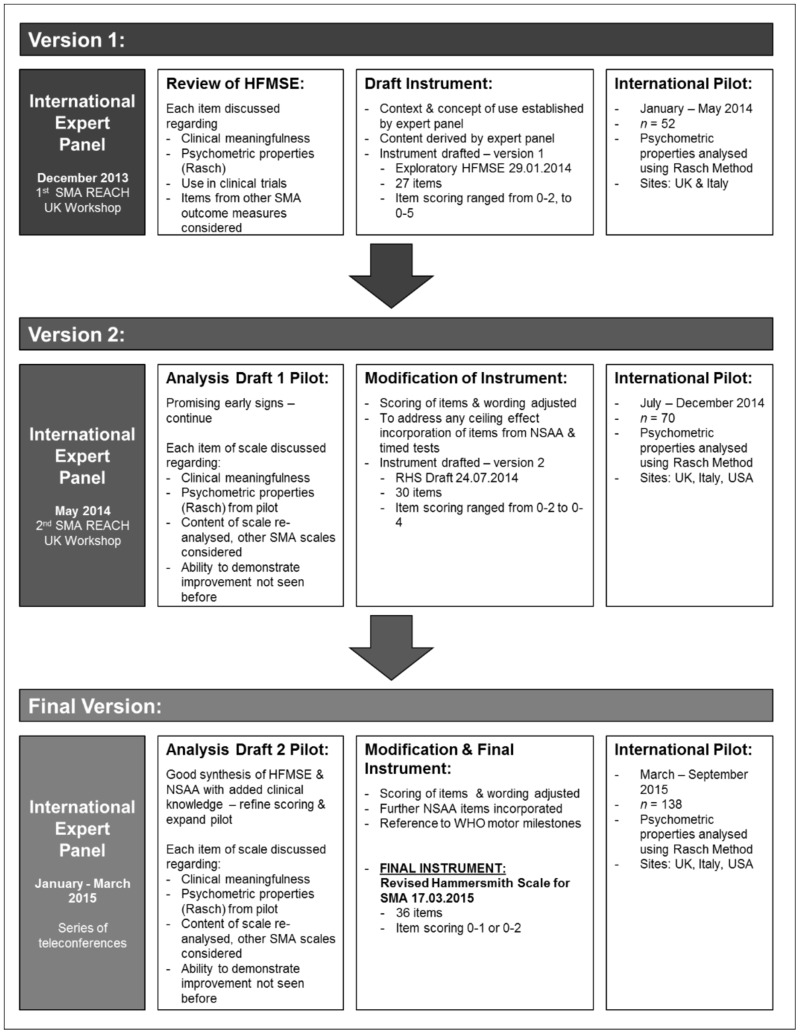
Revised Hammersmith Scale for Spinal Muscular Atrophy: Process of development. Iterative process of RHS development—expert panel, SMA outcome measure review, draft instrument, scale pilot and subsequent modification summarised for draft version 1, 2 and final version of the RHS.

An international multidisciplinary expert panel of Physiotherapists and Clinicians representing three established national networks, SMA REACH UK, the Italian SMA Network and the Paediatric Neuromuscular Clinical Research (PNCR) SMA Network USA, attended several in-depth workshops and teleconferences to revise the HFMSE (Fig 1). The intent was to develop a scale to assess the spectrum of gross motor function from weak individuals with type 2 SMA, who may have lost the ability to sit, through to strong ambulant individuals with type 3 SMA. The method of scale development followed recommendations by the United States Food and Drug Administration (FDA) for outcome measures [23]. Efforts were also made to ensure scale development met the criteria of the Consensus-based Standards for the selection of health status Measurement Instruments (COSMIN) checklist and in anticipation of the International Society for Pharmacoeconomics and Outcomes Research recommendations for good practice [24, 25].

At the time of revising the scale, priority was given to maintaining the original construct of the scale which has proved to be an excellent tool to monitor natural history and detect changes following clinical interventions [18, 26]. The use of Rasch methodology throughout the process cemented the intent for the scale to be more psychometrically robust. The expert panel was employed to ensure that the content and scoring of the scale, in addition to being psychometrically robust, was also relevant and applicable for use in everyday clinical practice to ensure it remained a scale with high clinical utility. The application of the scale was considered primarily an evaluative tool, however it was acknowledged it may have further potential to be both a discriminative and predictive tool, particularly with regards scale linearisation in the future. As with the original scales the need to remain aligned to the underpinning construct and use of minimal equipment was paramount.

#### Underpinning construct

The theoretical construct underpinning both the original and the revised scale was the natural history and pattern of weakness seen in SMA type 2 and 3 and its presentation during gross motor and functional activities. The scale was based upon a reflective conceptual model of motor performance in SMA. The intent was for the scale to reflect the development of functional mobility skills and include progressively more challenging activities, to ensure capacity for improvement could be demonstrated. The construct of interest is associated with the activity domain of the International Classification of Functioning, Disability and Health framework [27].

#### Clinical content validity—Definition of items

All 33 items of the HFMSE were discussed by the international expert panel regarding their individual clinical relevance in assessing physical abilities in SMA, their scoring criteria, psychometric properties and experience of use in clinical trials. The opportunity to expand the scale to a higher level of functional ability was also explored. Scoring criteria for each item was agreed to ensure each grade for the item represented a distinctly different change in function/ability; the application of modern psychometrics, Rasch analysis, aided this process. Commonly used compensations movements observed in SMA, in addition to the ability to achieve functional activities with/without compensations were discussed, and the ability to functionally achieve the item remained paramount. To distinguish between the more functionally able patients and to generate a scale with items charting the capacity to improve, the most difficult scoring criteria was often chosen as a grade which represented the abilities expected of the typically developing population. Where appropriate, experts drew on their clinical experience of functional scales used in SMA to reduce floor and ceiling effects, for example the Children’s Hospital of Philadelphia Infant Test of Neuromuscular Disorders (CHOP INTEND) and North Star Ambulatory Assessment (NSAA), to ensure the most relevant items for the target population were represented [28, 29]. The CHOP INTEND is a scale specifically designed to assess extremely weak infants with SMA type 1 [29]. The NSAA was originally developed as an outcome measure for the assessment of functional abilities in ambulant Duchenne Muscular Dystrophy and despite not having been formally validated for SMA, it has been shown to be a clinically relevant tool in the evaluation of ambulant SMA 3 patients [28, 30, 31].

Each expert panel workshop resulted in revisions to the scale, and the resultant scales were pilot tested in the international cohorts (Fig 1). In this paper we present the final version of the RHS. A manual of RHS testing procedures, detailed scoring criteria and testing proforma were produced in English for the UK and USA networks; these were then translated into Italian for the Italian Network by the Lead Italian Physiotherapist.

#### Psychometric properties—Rasch analysis

The HFMS and subsequent modifications were developed using classical test theory. It has been argued that scale development using traditional psychometric methods, due to their focus at the scale/test level, has limitations due to the inability to differentiate person ability and item difficulty, and such techniques provide a more gross estimate of reliability and standard error of measurement [32, 33]. Modern test theory, specifically latent trait theory (LTT), is widely advocated as a more detailed/sensitive and robust analytical approach to the assessment and development of improved health outcome measures [32–35].

Latent trait theories focus at the individual item and person level by addressing the relationship between the measurement and probability of their response occurring [35]. The modern psychometric technique (latent trait theory) employed in this study was the Rasch Unidimensional Measurement Model (unrestricted and simple logistic model); it uses five key tests of scale validity and reliability by addressing individual item fit and internal ordering within items, targeting, dependency, reliability and stability [21, 35, 36]. The method of estimation used was pairwise estimation for polytomous Rasch models [37]. The Rasch method is a robust approach and explores in detail why the data may not fit the mathematical model by testing both the stability of people and the instrument. It also allows rigorous testing of the construct of interest and thus employs theory-referenced measurement [35]. Rasch analysis was conducted using Rumm2030 software [38] to assess the psychometric properties of the two draft scales and the finalised RHS scale, this paper reports upon the final pilot of the RHS. Results from pilot testing earlier drafts informed re-design of the scale at each workshop/teleconference.

#### Additional statistical analysis

Descriptive statistics of median and interquartile range (IQR) were used to describe patient demographics. Discriminative and groups validity was assessed for ambulatory status, SMA type, SMA type and current ambulatory status combined, scoliosis surgery and highest current level of ability as assessed with the World Health Organisation (WHO) motor milestones [39]. If a participant achieved a score of 0 for the WHO motor milestones they were classified as unable to sit independently. The relationship with age was further analysed according to the stratification groups proposed by Mercuri et al., 2016 [18]; < 5 years, 5–14.9 years and ≥ 15 years. Groups and discriminant validity were assessed using the Kruskal Wallis and Mann-Whitney U tests, *p* < 0.05 was deemed significant and p ≤ 0.001 highly significant.

To test the construct of measuring progressively more difficult motor function ability, concurrent validity was assessed comparing the RHS with the WHO motor milestones using Spearman’s rho correlation [39]. Strength of correlations were quantified as moderate when *r*_*s*_ = 0.50 to 0.69, strong when *r*_*s*_ = 0.70 to 0.89 and very strong when *r*_*s*_ = 0.90 to 1.00. All additional analysis was conducted using IBM SPSS Statistics version 22 [40].

An enhanced utility of the RHS in the ambulant type 3 populations is the incorporation of two timed tests in item 19 –run/walk 10 metres and item 25 –rise from floor. A timed test subgroup analysis was conducted in type 3 ambulatory patients. Due to the relatively small subpopulation non-parametric tests were conducted and median and IQR presented, the relationship between age and total RHS score were investigated in addition to discriminative validity regarding ordinal scoring within the items.

#### Pilot testing the Revised Hammersmith Scale for SMA

The international consortium piloting the RHS consisted of three national networks comprising seven sites: SMA REACH UK—London and Newcastle, Italian SMA Network—Rome and the PNCR Network for SMA USA—Columbia, Philadelphia, Boston, and Stanford. Two draft revised scales were piloted prior to the final version: version 1 January—May 2014 (*n* = 52), and version 2 June to December 2014 (*n* = 70). Following each pilot the scale content, definition of items and scoring were re-analysed according to clinical meaningfulness and Rasch analysis and repeated until agreement was achieved on the final scale, the Revised Hammersmith Scale for SMA (RHS), in March 2015, Fig 1.

The final version of the RHS was piloted March to September 2015 alongside the WHO motor milestones. The WHO motor milestones are six clearly defined gross motor milestones recognised by WHO as essential to achieving independent ambulation [39]. The items assess sitting without support, hands and knees crawling, standing with assistance, walking with assistance, standing alone and walking alone. Completed alongside the RHS the WHO items were often more difficult than the equivalent RHS item. For example the RHS definition of independent sitting requires the individual to sit for a count of three compared to the WHO definition where sitting is maintained for ten seconds.

The participants assessed with the RHS were enrolled in network specific natural history studies which all had local ethical approvals in place permitting the pilot of functional scales (SMA REACH UK: National Research Ethics Committee (REC) London Bromley, Health Research Authority REC reference 13/LO/1748; PNCR USA Institutional Review Boards (IRB) and Numbers: Columbia University Medical Center Human Research Protection Office IRB reference AAAE8252, The Children’s Hospital of Philadelphia IRB reference 10–007816, Boston Children’s Hospital Office of Clinical Investigations IRB reference 05-02-028, Stanford University Research Compliance Office IRB reference 31140). The Italian ethical requirements for natural history studies mean that the Italian SMA Network was not provided with an IRB number. All participants had given their explicit written informed consent to participate in the site specific natural history studies. The evaluators conducting the RHS assessments were the expert physiotherapists who developed the scale in addition to network physiotherapists who were deemed competent to complete the RHS assessments following local training by the expert physiotherapists.

#### Additional reliability and validity testing

Content validity of the RHS from a patient/parent perspective in addition to preliminary inter and intra-rater reliability of the RHS in a UK cohort of Physiotherapists has been established in concurrent studies which will be reported separately in the future.

## Results

One hundred and thirty-eight patients with genetically confirmed chromosome 5q SMA classified with SMA type 2 or type 3 were evaluated using the RHS between March and September 2015 across the three national networks. Of the 138 patients 89 had type 2 and 49 had type 3, of these 40 were type 3a and 9 were type 3b. Ambulation was defined as the ability to ambulate without aids or orthotics over 10 metres, 65.3% (32/49) of patients with type 3 SMA were ambulant (type 3a *n* = 24, median age 9 years 10 months; type 3b *n* = 8, median age 18 years 7 months). There was similar distribution across the sexes with 72 males and 66 females assessed. The median age at assessment was 8 years 6 months (IQR 4 years 10 months to 12 years 4 months), the age range varied from 1 year 4 months to 51 years 7 months. With regards orthopaedic interventions, 10.1% of patients had undergone spinal surgery (*n* = 14).

### RHS content

The final RHS consisted of 36 items for very weak SMA 2 through to very strong SMA 3. With regards scoring, 33 items were graded on an ordinal scale of 0, 1, 2 where 0 denotes the least level of ability/function progressing to the highest level of ability to achieve a score of 2, the remaining 3 items were scored 0, 1 where 0 is unable and 1 was able to achieve. The maximum achievable score is 69. The scale was ordered to limit position change with items grouped according to position tested for example sitting, supine, prone, standing etc., items within that position progress from easier to more difficult i.e. items in supine move from crook/hook lying to lie to sit. To avoid a ceiling effect, the RHS contains revised items from the NSAA including two timed tests, and for the weaker end of the spectrum it also contains a revised item from the CHOP INTEND. Two RHS items incorporated the equivalent WHO motor milestone as their top score, item 16 cruising and item 18 walking. The current RHS scale proforma can be found in the supplementary information (S1 Appendix). The proforma includes the WHO motor milestones [39], which can be completed concurrently.

### RHS pilot

**Fig 2:**
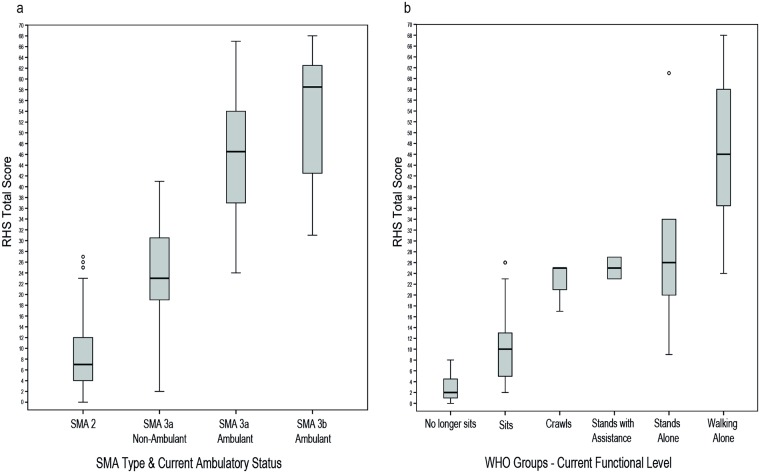
Discriminative/groups validity of the RHS. Median RHS total score (IQR and Range) versus a) SMA type combined with current ambulatory status; b) Highest current level of motor ability according to WHO groups.

**Fig 3:**
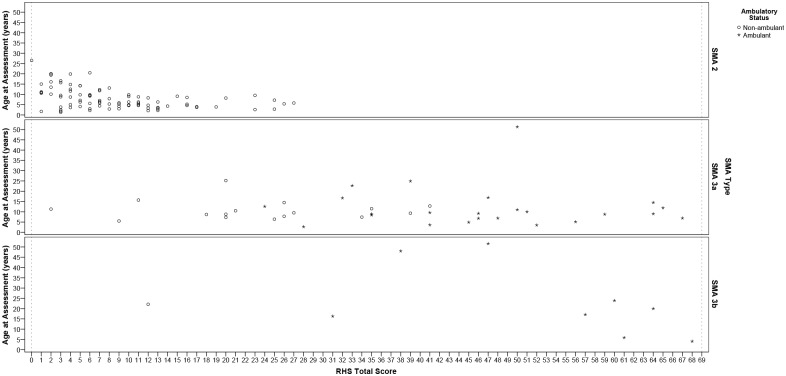
Individual RHS total score data points versus age and SMA type for entire pilot cohort (*n* = 138). RHS total score versus age and stratified according to SMA type, * ambulant and ° non-ambulant patients are distinguished, dotted lines represent floor (RHS total score = 0) and ceiling effect (RHS total score = 69).

The median score for the sample was 12 (IQR 6, 28), the lowest total score achieved on the RHS was 0 (*n* = 1) and highest was 68 (*n* = 1). The RHS was able to discriminate between clinically different groups (Table 1): SMA type (*p* < 0.001), ambulatory status (*p* < 0.001), SMA type and current ambulatory status combined (*p* < 0.001) Fig 2a, highest current level of functional ability as classified by WHO motor milestones (*p* < 0.001) Fig 2b, and whether a patient had spinal surgery or not (*p* = 0.001). Distinctly different ranges of scores within these groups was evident, Table 1, Fig 2. Gender did not have an influence on RHS scores (*p* = 0.986). The RHS was significantly strongly correlated with the WHO motor milestones *r*_*s*_ = 0.860, *p* < 0.001. Table 1 contains further details on participant scores and demographics, and refer to Fig 3 for the complete dataset scatterplot of RHS total scores versus age, stratified according to SMA type with ambulant and non-ambulant patients distinguished.

**Table 1:** RHS Pilot sample demographics and discriminative/groups validity.

		n	Median Age years (IQR)	Median RHS Score (IQR)	Range	Groups validity(*p* value)
**All participants**		138	8.5 (4.8, 12.3)	12 (6, 28)	0–68	
**SMA Type**	2	89	6.3 (4.2, 10.1)	7 (4, 12)	0–27	< 0.001*^a^
	3a	40	9.3 (7.1, 12.7)	37 (26, 49)	2–67	
	3b	9	20 (16.3, 23.9)	57 (38, 61)	12–68	
**Ambulatory Status**	Non- Ambulant	106	7.4 (4.6, 11.2)	9 (4, 15)	0–41	< 0.001*^b^
	Ambulant	32	9.8 (6.9, 17)	48 (39, 60)	24–68	
**SMA Type & Current Ambulatory Status**	2	89	6.3 (4.2, 10.1)	7 (4, 12)	0–27	< 0.001*^a^
	3a non-ambulant	16	9.4 (7.6, 12.2)	23 (19, 31)	2–41	
	3b non-ambulant	1	22.1	12	12	
	3a ambulant	24	9.1 (6.9, 13.6)	47 (37, 54)	24–67	
	3b ambulant	8	18.6 (11.1, 36.0)	59 (43, 63)	31–68	
**WHO Groups—Current Functional Status**		131	7.9 (4.6, 11.8)	12 (5, 27)	0–68	< 0.001*^a^
	No longer sits	16	11.1 (7.8, 15.6)	2 (1, 5)	0–8	
	Sits	71	6.3 (4.2, 9.8)	10 (5, 13)	2–26	
	Crawls	4	5.1 (3.3, 6.8)	25 (21, 25)	17–25	
	Stands with assistance	2	4.2 (2.6, 5.8)	25 (23, 27)	23–27	
	Walks with assistance	1	9.5	27	27	
	Stands alone	5	7.4 (5.9, 7.8)	26 (20, 34)	9–61	
	Walks alone	32	9.8 (6.9, 16.8)	46 (37, 58)	24–68	
**Gender**	Male	72	8.1 (4.9, 11.6)	12 (6, 29)	1–67	0.986^b^
	Female	66	8.6 (4.8, 12.6)	12 (6, 28)	0–68	
**Spinal Surgery**	No	124	7.3 (4.6, 11.1)	13 (7, 33)	0–68	0.001*^b^
	Yes	14	13.3 (11, 16.1)	3 (2, 6)	1–50	

*Highly significant *p* ≤ 0.001

^a^ Kruskal Wallis,

^b^Mann-Whitney U Test

Overall no relationship was observed between age and RHS score *r*_*s*_ = 0.021 (*p* = 0.805), a statistically significant inverse relationship with age was observed in the type 2 population *r*_*s*_ = -0.451 (*p* < 0.001), this was also observed to a lesser extent in the non-ambulant population *r*_*s*_ = -0.242 (*p* = 0.007), however both of these were deemed to be moderate to low correlations. When stratifying the whole population into age groups of those < 5 years, 5–14.9 years and ≥ 15 years no statistically significant differences in RHS score were observed across these groups (*p* = 0.832). Further analysis of these age groups versus SMA type identified a pattern of declining scores as age increased within type 2 SMA subjects (Kruskal Wallis *p* < 0.001), Table 2. This was not statistically different in the type 3 population (SMA 3a *p* = 0.619, SMA 3b *p* = 0.187), however an overall trend with declining scores as age increased was observed. With regards ambulatory status a statistically significant pattern of declining scores as age increased was observed in the non-ambulant population (*p* = 0.007), Table 2.

**Table 2:** Age stratification versus median RHS score (IQR).

	n	< 5 years	n	5–14.9 years	n	≥ 15 years	Groups validity (*p* value)^a^
**Total population**	36	12 (7, 17)	78	11 (6, 27)	24	16 (3, 43)	0.832
**SMA 2**	31	10 (6, 13)	48	7 (5, 11)	10	2 (2, 3)	< 0.001**
**SMA 3a**	4	43 (35, 49)	29	35 (25, 50)	7	33 (20, 47)	0.619
**SMA 3b**	1	68	1	61	7	47 (31, 60)	0.187
**Non-ambulant**	31	10 (6, 13)	62	9 (5, 20)	13	3 (2, 6)	0.007*
**Ambulant**	5	45 (41, 52)	16	51 (44, 63)	11	47 (33, 57)	0.463

*significant,

**highly significant,

^a^Kruskal Wallis

### Psychometric properties of RHS

**Fig 4:**
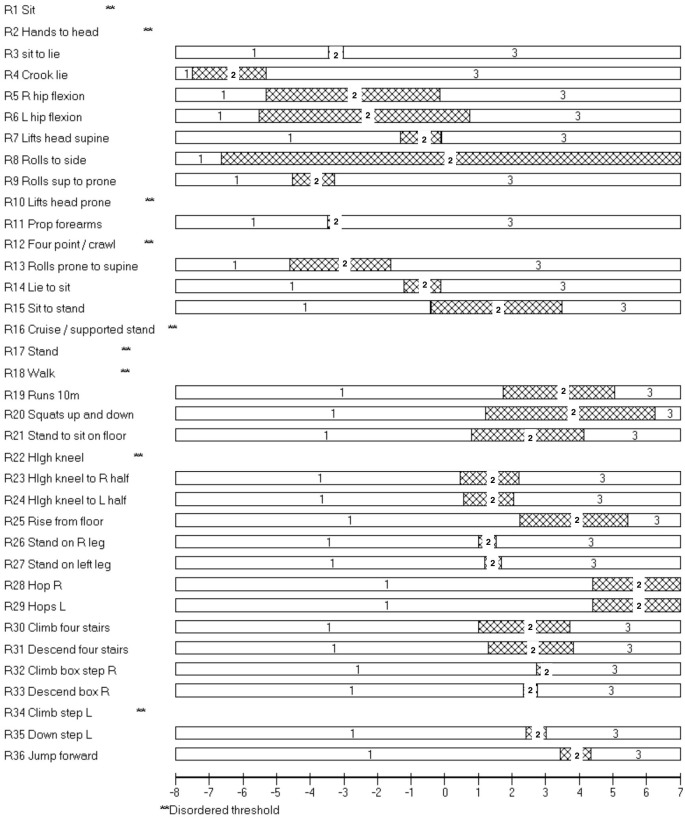
Rasch analysis: RHS 17.03.2015 threshold map for items in RHS in ranked order of difficulty. The presence of horizontal bars indicates that for these items as an individual’s ability increases they would be more likely to achieve a higher score and that this would increase systematically in a logical progression. They would first score 0, then 1 and then 2 as ability improves. The inverse is also true. Within each bar a number 1 represents a score of 0 on the RHS, 2 represents a score of 1, and 3 represents a score of 2.

**Fig 5:**
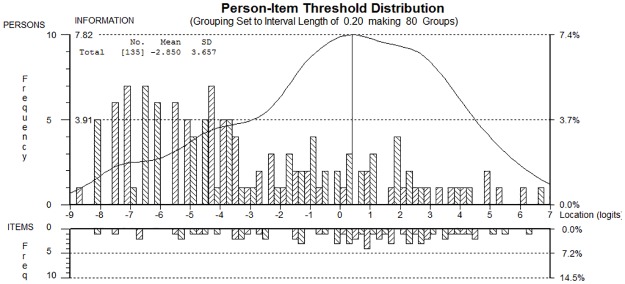
Rasch analysis: RHS 17.03.2015 person item threshold distribution. **Targeting of the patient sample (top) to individual items (bottom)**. The figure shows the targeting between the distribution of person measurements (upper histogram) and the distribution of the item locations (lower histogram).

Of the 138 assessments there were 3 invalid results and one extreme score resulting in 134 assessments entered into the Rumm2030 software. Overall there was very good fit of the 36 items to the construct of motor performance in SMA, with no items with a fit residual outside of ±2.5 (all items observed fit the predicted model well) and only one item, supine to side lying (item 8), had a significant χ^2^ probability (*p* < 0.001; Table 3). A significant value is an indicator that supine to side-lying is the item that fits the overall concept of motor ability in SMA least well. Good reliability was demonstrated by a high Person Separation Index (PSI) 0.98 (Table 4). There were logical and hierarchical individual item scores for 27/36 items (Fig 4). The targeting of the items was excellent with minimal ceiling effect (Fig 5), however there were fewer items measuring the ability of the weaker non-ambulant patients. Dependency was noted between the items which assess both right and left and similar items such as rolling from prone to supine and supine to prone. Removing items which were repeated (removed left side) did not influence the PSI (0.98) which remained virtually the same. Unidimensionality was acceptable (t-test 7.3%, binomial test lower 95% confidence interval proportion, 0.05).

**Table 3:** Individual item fit for RHS in order of difficulty.

Seq	Item	Location	Fit Residual	Chi Squared	Chi squared probability
**1**	Sit	-7.405	-0.035	0.658	0.7196
**8**	Supine to side lying	-6.647	0.085	20.584	0.0000*
**4**	Crook lying	-6.409	0.043	8.18	0.0167
**2**	Hands to head	-4.453	-0.022	1.89	0.3887
**9**	Rolls supine to prone	-3.909	-0.281	5.07	0.0793
**11**	Props on forearms	-3.385	-0.422	1.211	0.5459
**3**	Sit to lie	-3.247	-0.339	1.064	0.5875
**13**	Rolls prone to supine	-3.104	-0.757	5.047	0.0802
**5**	R hip flexion	-2.73	1.568	12.294	0.0021
**6**	L hip flexion	-2.386	2.496	8.413	0.0149
**10**	Lifts head from prone	-2.016	0.195	5.887	0.0527
**12**	Four point/ crawl	-1.155	-0.278	0.442	0.8018
**16**	Cruise / supported stand	-1.108	-0.933	1.517	0.4683
**7**	Lifts head supine	-0.717	1.103	9.377	0.0092
**14**	Lie to sit	-0.669	-0.686	0.645	0.7245
**17**	Standing	-0.226	-0.529	0.8	0.6704
**18**	Walking	0.127	0.075	3.808	0.1490
**22**	High kneeling	0.564	-0.319	2.067	0.3557
**26**	Stand on R leg	1.263	-0.507	1.135	0.5669
**24**	High kneel to L half	1.306	-0.405	2.511	0.2850
**23**	High kneel to R half	1.328	-0.559	2.397	0.3017
**27**	Stand on L leg	1.436	-0.452	0.975	0.6142
**15**	Sit to stand	1.533	-1.015	4.634	0.0986
**30**	Climb stairs	2.357	-0.662	1.13	0.5682
**21**	Stand to sit on floor	2.477	-0.432	0.118	0.9426
**33**	Down box step R	2.549	-0.244	0.428	0.8076
**31**	Descend stairs	2.555	-0.301	0.828	0.6609
**35**	Down box step L	2.716	-0.235	0.514	0.7734
**32**	Climbs box step R	2.831	-0.254	0.632	0.7289
**34**	Climbs box step L	2.857	-0.217	0.815	0.6655
**19**	Runs 10 metres	3.401	-0.398	0.672	0.7145
**20**	Squat up and down	3.735	-0.478	0.912	0.6338
**25**	Rise from floor	3.828	-0.264	0.326	0.8495
**36**	Jumps forward	3.896	-0.125	0.105	0.9605
**28**	Hops R	4.401	-0.156	0.32	0.8593
**29**	Hops L	4.407	-0.157	0.32	0.8591

* significant χ^2^ probability p = 0.001

**Table 4:** Overall properties of RHS using the Rasch measurement method.

	Item Fit	SD	Person Fit	SD	PSI	DF
**RHS**	-0.164	0.658	-0.226	0.337	0.9753	72

PSI—Person separation index; DF—Degrees of freedom

#### Disordered thresholds

Disordered thresholds are often highlighted during Rasch analysis and denote items where the scoring grades are not distinctly different from one another, therefore meaning that those items are not working from a psychometric perspective. Nine disordered thresholds (Fig 4) were observed in the following items: sitting, hands to head in sitting, lifting head from prone, four-point kneeling/crawling, cruising/supported stand, standing, walking, high kneeling and climb step left. In all cases the middle score of ‘1’ was not being detected as being distinctly different.

### Timed test subgroup analysis: Items 19 and 25

Of the 49 type 3 patients, 13 with type 3a also completed the timed aspects of the 10 metre run/walk (item 19) and 14 with type 3a completed the timed rise from floor (item 25). In both groups age was not correlated with the overall RHS total score, Table 5 (timed 10 metre group *r*_*s*_ = -0.079 *p* = 0.797; timed rise group *r*_*s*_ = -0.011, *p* = 0.971). A significant inverse linear relationship between RHS total score and timed tests was found (Table 5); 10 metre run *r*_*s*_ = -0.912 strong correlation *p* < 0.001, rise from floor *r*_*s*_ = -0.693 moderate correlation *p* < 0.001. Both timed tests were moderately correlated with each other, however this did not achieve statistical significance *r*_*s*_ = 0.588 *p* = 0.074, Table 5. The ordinal scoring utilised in both item 19 and 25 enabled further discrimination within the timed tests by distinguishing between clinically different abilities *p* < 0.05 in all cases, Table 5.

**Table 5:** SMA Type 3 sub analysis: RHS timed tests (all SMA 3a).

Timed 10 m (n = 13)				Timed Rise from floor (n = 14)			
	Median (IQR)	Correlation with timed test *r*_*s*_ (*p* value)^a^	Discriminative Validity *p* value^b^		Median (IQR)	Correlation with timed test *r*_*s*_ (*p* value)^a^	Discriminative Validity *p* value^b^
**Age (years)**	9 (6.9, 11.9)	0.049 (0.873)		**Age (years)**	9.2 (6.9, 11.9)	0.336 (0.240)	
**RHS Total Score**	51 (48, 59)	-0.912 (< 0.001**)		**RHS Total Score**	50 (39, 59)	-0.693 (0.006*)	
**Item 19: 10 metre Run (secs)**	8.53 (5.62, 9.73)	-0.741 (0.004*)		**Item 25: Rise from Floor (secs)**	7.8 (4.03, 18.33)	-0.703 (0.005*)	
**Item 19 Score 0 (secs)**	15.52 (9.73, 21.30)		0.035*	**Item 25 Score 0 (secs)**	18.33 (15.57, 18.81)		0.039*
**Item 19 Score 1 (secs)**	8.53 (6.02, 9.44)			**Item 25 Score 1 (secs)**	5.18 (3.70, 7.80)		
**Item 19 Score 2 (secs)**	4.48 (4.47, 4.50)			**Item 25 Score 2 (secs)**	3.58 (3.58, 3.58)		

^a^Spearman’s Rho,

^b^Kruskal Wallis,

*Significant p < 0.05,

**Highly significant p ≤ 0.001.

## Discussion

The original HFMSE has clear clinical utility in the assessment of SMA and is being successfully used in ongoing studies. Nevertheless its psychometric properties have demonstrated some discontinuities. In addition, ongoing therapeutic developments highlight the need for a scale which has the capacity to demonstrate improvement, and in this respect the HFMSE may be more susceptible to ceiling effects in stronger patients following successful therapeutic intervention. We have undertaken a comprehensive process to produce an SMA specific motor performance rating scale which is robust from a psychometric perspective, clinically relevant and with the capacity/sensitivity to demonstrate improvement.

We have established in an international multicentre study that the RHS is able to assess a broad range of physical abilities across the spectrum, from weak SMA type 2 through to very strong type 3. The scale therefore demonstrates face validity of the underpinning concept of interest and construct of the RHS. The widespread scatter of scores observed in the SMA 3a range (Fig 3) is of particular interest because it demonstrates the ability of the scale to capture the continuum from ambulant to non-ambulant, and it could be postulated that the converse could also be true. This together with the lack of ceiling effect (68 was the maximum score achieved *n* = 1) and inclusion of timed tests for type 3 patients highlights that the RHS possesses the capacity to demonstrate further improvement in both SMA types 2 and 3.

The RHS correlated strongly with the WHO motor milestones confirming the scale measures progressively more difficult motor abilities, therefore establishing concurrent validity of the scale. The RHS demonstrated discriminative groups validity by statistically distinguishing between clinically different groups: current WHO functional ability, SMA type, ambulatory status, SMA type combined with current ambulatory status and whether patients underwent surgery or not. Further longitudinal studies are required to investigate discriminant validity with regards markers of SMA disease pathology for example SMN2 copy number and CMAP. Content validity from a patient perspective and inter and intra-rater reliability has been studied in the UK and is presented in parallel papers currently in preparation. Further international inter and intra-rater reliability testing is planned.

Rasch analysis demonstrated that the RHS measures with a good degree of accuracy the construct of motor performance in type 2 and 3 SMA. The strength of the RHS is the psychometrically ordered scoring determined through the application of clinical sensibility. This has resulted in increased items which sensitively capture a broader spectrum of abilities. The scoring system worked well in 27/36 items, the remaining 9 items were classified as having disordered thresholds whereby the middle score of 1 was not being picked up as being distinctly different to a score of 0 or 2. The majority of disordered thresholds occurred in what could be considered ‘transitional items’ relevant in specific SMA subtypes where a patient may be considered anecdotally as a ‘weaker type 2’, ‘strong type 2’, ‘weaker type 3’ or in the process of transitioning from ambulant to non-ambulant. These subgroups were represented by a small number of patients. This study used a cross-sectional sample to test the RHS, and it is possible that these ‘transitional items’ may be more relevant in a longitudinal cohort to assess change within individual subjects. Over time these items may, for example, capture the transient physical abilities observed during the progression from ambulant to non-ambulant or when losing the ability to sit. It would be difficult to gather a sufficient sample of patients to demonstrate this cross-sectionally, and whilst the transitional items represent a small proportion of patients, the consensus of the experts was that there is definite clinical value in capturing this information. Transitional items are also important when considering clinical trials. There may be potential treatments for SMA which may demonstrate an improvement in the condition not seen previously in the natural history, for example acquisition of skills progressing from non-ambulant to ambulant. Therefore, despite the relative inconsistencies observed in the nine disordered thresholds, the expert panel recommended retention of these items with their current scoring criteria due to their clinical utility and potential to more sensitively capture longitudinal progression or potential improvement in SMA over time. Longitudinal changes at six and twelve months are currently being investigated.

The relationship between age and RHS score was most apparent when looking at the stratified age groups < 5 years, 5–14.9 years and ≥ 15 years in SMA 2 patients and non-ambulant patients. These results are in keeping with those found by Mercuri et al., (2016) whereby the influence of age on longitudinal HFMSE functional scores was greatest in non-ambulant patients suggesting a different trajectory of progression when compared with ambulant patients at both baseline and 12 months [18].

Since the introduction of the classification of the three classical types of SMA observed in infancy/childhood there has been discussion as to whether this approach is too rigid given the wide spectrum of physical ability observed within each type [41–44]. The original HFMS paper proposed criteria to aid sub classifying SMA type 2 into ten further decimalised categories based upon the score achieved on the HFMS, although used anecdotally in highly experienced clinicians this has not to date been adopted by the SMA community as a whole [10]. The last few years has seen a concerted effort to improve the descriptors for SMA types 0, 1 and 3 with further sub-division of type often noted with a letter i.e. SMA 1b, 3a etc. [9, 10, 41]. In this study the WHO motor milestones were used to identify the current level of function in SMA patients, this may be different to the highest level of ability ever achieved required for classifying SMA type, for example a patient who was classified as type 2 who has lost the ability to sit. The WHO motor milestones are more detailed functional descriptors in comparison to SMA type or current ambulatory status, but are not as comprehensive in describing the current functional abilities as the RHS itself. Using the WHO and RHS together highlighted further sub-populations and distinct ranges of functional scores for certain abilities, for example those who are no longer able to sit scored between 0 and 8 on the RHS, those who crawled score between 17 and 24, and those who walked scored between 24 and 68. These, as preliminary findings of a new scale, require further validation in large longitudinal multi-centric studies. Revisiting use of a more sensitive classification by using the WHO motor milestones and the RHS together may be a useful strategy to aid understanding and future stratification of ‘within type’ SMA natural history trajectories. This may lead to the development of more refined trial inclusion/exclusion criteria. A recommendation following this study would be to expand the qualifiers to include the ability to ambulate independently (thus be qualified as ambulant) without orthotics or aids over 10 metres, this information could be somewhat extrapolated from RHS item 19.

Recently the need for items which are able to sensitively discriminate between groups of patients has become even more pertinent [45, 46]. An increased utility of the RHS in comparison with other outcome measures currently used in clinical trials is the inclusion of timed tests and discriminative items for type 3 patients. This study identified that the individual item ordinal scores for the timed test items 19 and 25 can discriminate between clinically different groups in SMA type 3a patients in the times they achieved. Although these milder individuals only represented a small number of patients, this highlights a further discriminative capability of the RHS which is advantageous and warrants further investigation.

In this study the impact of contractures, scoliosis, height and weight on the functional score was not investigated, further studies are required to investigate the extent to which these factors may have a confounding impact on the RHS. Further work is required regarding the potential for the RHS to act as a transitional scale for stronger type 1 patients when the CHOP INTEND scale is no longer appropriate and a floor effect exists on the HFMSE, this is particularly pertinent in light of the ongoing clinical trials in this patient population.

## Conclusion

This study has described in comprehensive detail the content, construct, concurrent and discriminative groups validity properties of the RHS scale in a large international cohort of patients with SMA types 2 and 3. The strength of the RHS is that it has been developed to address the minor discontinuities observed in the original HFMSE and has expanded the ability to monitor changes at the two extremes of the scale which is particularly relevant for translational research applications. It has been prospectively designed using rigorous clinical reasoning and modern psychometrics, therefore complementing existing constructs making it a valid instrument for clinical trials.

Use of the RHS in combination with the WHO motor milestones may enable more sensitive description of SMA phenotype and trajectories, which may in turn, facilitate more accurate sub-type analysis. Our findings also suggest that the incorporation of timed tests may enable further discrimination within type 3 patients. Further work is needed to establish how the RHS should be used in conjunction with the CHOP INTEND for very weak infants and to establish the relationship with Upper Limb Modules or person reported outcome measures. Work is underway to investigate the clinical meaningfulness of the nine items with disordered thresholds, and longitudinal investigation of the RHS measurement properties over six and twelve months. With regards psychometric properties it is anticipated that a linearised version of the scale will be developed, which will be underpinned by the parallel work undertaken to address content validity from a patient perspective.

One of the key strengths of the RHS is the rigorous iterative process employed during its development, expanding upon an already well-developed scale, involving use of expert panels, psychometric analysis and several international pilots, resulting in construction of a robust SMA specific clinical outcome assessment tool. As the results demonstrate, the RHS is a versatile tool able to capture a broad range of abilities across the spectrum of SMA, from young children through to adults, and in varying stages of the disease course.

## Supporting information

S1 Appendix RHS Testing proforma version 17.03.2015 including WHO motor milestones. (PDF)
