# Supplementary material for: Revised Hammersmith Scale for spinal muscular atrophy: A SMA specific clinical outcome assessment tool
Source: PLoS One. 2017 Feb 21;12(2):e0172346. doi: 10.1371/journal.pone.0172346 (PMC5319655; doi:10.1371/journal.pone.0172346)
Supplement: S1 Appendix — (PDF) [file pone.0172346.s001.pdf]

Revised Hammersmith Scale for SMA (RHS) 17.03.2015

Name **SMA Type** **DOB** **Assessor**
Date of assessment **Albuterol/Salbutamol Y / N** **Date of spinal surgery**

Please circle highest current level of independent mobility:

None Rolls Bottom shuffles Crawls Walks with KAFO's / AFO's Walks with crutches / frame Independent walking

Comment………

LBC = Limited by contracture *WHO separate scoring at end of RHS

	Test	*Instruction*	2	1	0	LBC	Comments	RHS Score
SITTING	**1* Sitting** Using plinth / floor. *(Back / feet unsupported)*	*Can you sit on the plinth/chair without using your hands for support for a count of 3?*	Sitting unsupported: Ring or 90/90	Maintains seated position via propping with hand/s: Ring or 90/90	Unable to sit		**WHO 1*** ☐ Long sitting	
SITTING	**2 Hands to head in sitting**	*Can you show me how you get your hand/hands to your head?* *(hands touch head above level of ears/ eyebrows)*	Able to bring both hands to head at same time – arms free from side without flexing head or trunk	Able to bring one hand to head – arms free from side without flexing head or trunk ☐ R ☐ L	☐ Using compensations – flexing head & trunk or "crawling" hand/s ☐ Unable to bring hand to head		Sitting position described: ☐ 90/90 ☐ Ring ☐ Long	
SITTING	**3 Sitting to lying**	*Can you lie down in a controlled/safe way from sitting?*	Able to lie down through side lying or using clothes in a controlled/safe way	Able to lie down by going forwards and rolling sideways, or through prone in a controlled/safe way	Unable or completes in uncontrolled/ unsafe way			
SUPINE	**4 Adduction from crook (hook) lying –** supine hips @ 45°, knees @ 90°, feet hip width apart. Passively abduct leg	*Can you bring your leg back to the middle and hold this position for a count of 3?*	Able to adduct to bring 1 leg back to neutral	Holds crook lying position for a count of 3	Unable to maintain/ achieve starting position.		☐ R ☐ L	
SUPINE	**5 Right hip flexion in supine**	*Can you bring your right knee to your chest? Try to go as far as you can*	Full hip flexion achieved	Initiates right hip and knee flexion (>10% of available range of motion)	Unable		Flexion contracture > 15 ° present? Hip Y ☐ N ☐ Knee Y ☐ N ☐	
SUPINE	**6 Left hip flexion in supine**	*Can you bring your left knee to your chest? Try to go as far as you can*	Full hip flexion achieved	Initiates left hip and knee flexion (>10% of available range of motion)	Unable		Flexion contracture > 15 ° present? Hip Y ☐ N ☐ Knee Y ☐ N ☐	
SUPINE	**7 Lifts head from supine**	*Can you lift your head to look at your toes keeping your arms folded for a count of 3?*	Can lift head up through neck flexion and holds for a count of 3	☐ Can lift head with compensatory movements for a count of 3 ☐ Can lift head up momentarily through neck flexion (< a count of 3)	Unable			
ROLLING	**8 Supine to side-lying** (end position leading leg ends up on top of other)	*Can you roll onto your side?* Note: Onto side = shoulders perpendicular to floor, trunk and hips in line with body		Able to roll onto side	Unable to roll onto side in any direction		☐ R ☐ L	
ROLLING	**9 Rolls supine to prone**	*Can you roll from your back to your tummy? Try not to use your arms*	Rolls fully into prone without pulling/ pushing on arms	Rolls fully into prone by pulling/pushing on arms	Unable to roll into prone in any direction. Does not initiate or complete		☐ R ☐ L	
PRONE	**10 Lifting head from prone**	*Can you lift your head up keeping your arms by your side for a count of 3*	Lifts straight up, arms down by sides for a count of 3	Lifts straight up arms forward for a count of 3 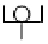	Unable or lifts less than a count of 3			
PRONE	**11 Prone; prop on forearms**	*Can you prop yourself on your forearms with your head up (not holding your head) For a count of 3*	Achieves position and holds head up independently for a count of 3	Maintains position with head supported on hands for a count of 3	Unable or holds for less than a count of 3			
PRONE	**12* Four-point kneeling/ crawling**	*Can you get onto your hands and knees and then crawl?*	Crawls moving all 4 limbs at least 2 times in a row	Achieves four-point kneeling	Unable		**WHO 2***	
ROLLING	**13 Rolls prone to supine**	*Can you roll from your tummy to your back? Try not to use your arms*	Rolls fully into supine without pulling/ pushing on arms	Rolls fully into supine by pulling/pushing on arms	Unable to roll into supine in any direction. Does not initiate or complete		☐ R ☐ L	

	Test	Instruction	2	1	0	L B C	Comments	RHS Score
SITTING	**14 Lying to sitting**	*Can you get from lying (supine) to sitting?*	Able via supine / side lying using:<br>☐ 1 hand<br>☐ 2 hands	Able by using strategies:<br>☐ Turns into prone or towards floor<br>☐ Utilises momentum/ pulls on both legs	Unable			
	**15 Sit to Stand** (Starting in a 90/90 position using a Plinth / Bench / Chair)	*Can you stand up from the chair / bench keeping your arms folded if you can?*	Able to stand up without using arms (keeping arms folded, feet do not move)	Stands by using strategies: (circle relevant)<br>Uses hands on legs/chair<br>Turns Prone / Widens Base	Unable			
STANDING	**16* Cruising/ supported stand**	*Can you walk holding on around furniture?*	Cruise at least 5 steps around furniture<br>☐ R ☐ L	Stands supported for a count of 3	Unable to stand supported		**WHO 3 & 4***	
	**17* Standing**	*Can you stand without holding onto anything for a count of 3?*	Stands <u>upright</u> and <u>symmetrically</u>, without compensation for a count of 3	Stands but with some degree of compensation for a count of 3	Cannot stand independently, needs support		**WHO 5***	
RUN / WALK	**18* Walking**	*Can you walk without using any help or aids? Show me*	Takes at least 5 steps unaided	Able to take < 5 steps unaided	Unable		**WHO 6***	
	**19 Runs 10m**	*Can you go as quickly and safely as you can to……(give 10 m marker) "Ready steady GO"*	Can run - both feet off the ground	Speeds up walk but always maintains one foot on the floor	☐ Walks with no extra speed<br>☐ Only with aids<br>☐ Unable		**TIME:**…………seconds	
STANDING	**20 Squat down and up**	*Can you squat down and stand up again?*<br>*Pretend you are going to sit on a very low seat - only go as far down as you are able to get up from on your own*	Full squat down (without using hands) and up* (with/without using hands)<br>At least 90° achieved at knees/ hips<br>*must be able to get up from final range	Initiates squat in both knees (10° to <90°) with controlled movement +/- using hands on self<br>☐ Using hands	Unable			
	**21 Stand to sit on floor**	*Can you sit on the floor in a controlled/ safe way from standing?*<br>*Try not to use your arms*	Able to sit down arms free in a controlled manner	Sits on floor using hands on floor/body	☐ Uses furniture<br>☐ Unable			
MAT	**22 High kneeling**	*Can you kneel like this (high kneeling) for a count of 10?*	Maintains high kneeling without holding on for a count of 10	Maintains high kneel with one arm support for a count of 10	Unable			
	**23 High Kneeling to <u>Right</u> half kneel**<br>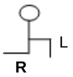<br>**(Left foot forward)**	*Can you bring your LEFT leg up so that your foot is flat on the ground without using your arms and hold for a count of 10?*	Able to achieve and maintain position without arm support for a count of 10	Maintains half kneel with one arm support for a count of 10<br>With:<br>☐ support<br>☐ no support	Unable			
	**24 High Kneeling to <u>Left</u> half kneel**<br>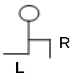<br>**(Right foot forward)**	*Can you bring your RIGHT leg up so that your foot is flat on the ground without using your arms and hold for a count of 10?*	Able to achieve and maintain position without arm support for a count of 10	Maintains half kneel with one arm support for a count of 10<br>With:<br>☐ support<br>☐ no support	Unable			
MAT / FLOOR	**25 Rise from floor**	*Can you get up from the floor using as little support as possible and as fast as you can?*<br>*Stand up as quickly as possible when the instruction "GO" is given*	Achieves rise from floor without hands (any unsupported method)	Able to get up from floor using hands on floor and/or body	☐ Able to get up from floor using furniture/ assistance<br>☐ Unable to get up from floor using any method		If uses ½ kneel:<br>☐ R (L foot forwards)<br>☐ L (R foot forwards)<br>Further detail:<br>**TIME:**……….seconds	
SINGLE LEG STAND	**26 Stand on one leg – Right**	*Can you stand on your right leg for a count of 3?*	Able to stand in a relaxed manner (no fixation) for a count of 3	Stands but either momentarily or needs a lot of fixation e.g. by knees tightly adducted or other trick	Unable			
	**27 Stand on one leg – Left**	*Can you stand on your left leg for a count of 3?*	Able to stand in a relaxed manner (no fixation) for a count of 3	Stands but either momentarily or needs a lot of fixation e.g. by knees tightly adducted or other trick	Unable			
	**28 Hops – Right**	*Can you hop on your right leg?*		Distinct hop: clears forefoot and heel off floor	Unable			
	**29 Hops – Left**	*Can you hop on your left leg?*		Distinct hop: clears forefoot and heel off floor	Unable			

	Test	Instruction	2	1	0	LBC	Comments	RHS Score
STAIRS	**30 Ascend stairs** 4 standard stairs	*Can you walk up the steps?* *If support is needed you may only use one rail (can use two hands to one rail)*	Independently (without support/rail) ascends using alternating pattern	☐ Alternating pattern with one support (one rail/hand on body) ☐ Marking time with/without support	Unable			
	**31 Descend stairs** 4 standard stairs	*Can you walk down the steps?* *If support is needed you may only use one rail (can use two hands to one rail)*	Independently (without support/rail) descends using alternating pattern	☐ Alternating pattern with one support (one rail/hand on body) ☐ Marking time with/without support	Unable			
STEP	**32 Climbs box step – Right**	*Can you step onto the top of the box using your right leg first?*	Faces forwards, climbs up – no support needed	Goes up sideways or needs support	Unable			
	**33 Descends box step – Right**	*Can you step down from the box using your right leg first?*	Faces forward, climbs down controlling weight bearing leg. No support needed	Goes down sideways or skips down or needs support	Unable			
	**34 Climbs box step – Left**	*Can you step onto the top of the box using your left leg first?*	Faces forwards, climbs up – no support needed	Goes up sideways or needs support	Unable			
	**35 Descends box step - Left**	*Can you step down from the box using your left leg first?*	Faces forward, climbs down controlling weight bearing leg. No support needed	Goes down sideways or skips down or needs support	Unable			
JUMP	**36 Jump forwards 30cm (12")**	*Can you jump as far as you can, with both feet, from this line all of the way to the other line?*	Jumps forward and lands with two feet simultaneously moving forward at least 30cm (12")	☐ Jumps with two feet simultaneously but lands one at a time moving forward/up travelling < 30cm (12") ☐ Jumps and lands with two feet simultaneously moving forward < 30cm (12")	Unable to initiate jump			
			**2's =**	**1's =**	**0's =**		**Total =**	**/69**

*WHO DEVELOPMENTAL MILESTONES	Qualifier met:
**W1. Sitting without support:** : Child sits upright with head erect for at least 10 seconds. Child does not use arms or hands to balance body or support position.	Y☐ N☐
**W2. Hands and knees crawling:** Child alternately moves forward or backward on hands and knees. The stomach does not touch the supporting surface. There are continuous and consecutive movement, at least 3 in a row.	Y☐ N☐
**W3. Standing with Assistance:** Child stands in upright position on both feet, holding onto a stable object (e.g. furniture) with both hands without leaning on it. The body does not touch the stable object, and the legs support most of the body weight. Child thus stands with assistance for at least 10 seconds.	Y☐ N☐
**W4. Walking with Assistance:** Child is in upright position with the back straight. Child makes sideways or forward steps by holding onto a stable object (e.g. furniture) with one or both hands. One leg moves forward while the other supports part of the body weight. Child takes at least 5 steps in this manner.	Y☐ N☐
**W5. Standing Alone:** Child stands in upright position on both feet (not the toes) with the back straight. The legs support 100% of the child's weight. There is no contact with a person or object. Child stands alone for at least 10 seconds.	Y☐ N☐
**W6. Walking Alone:** Child takes at least 5 steps independently in upright position with the back straight. One leg moves forward while the other supports most of the body weight. There is no contact with the person or object.	Y☐ N☐
**Total WHO Milestones Achieved:**	**/ 6**

Revised Hammersmith Scale for SMA (RHS) 17.03.2015

Is this test a true reflection of the subjects ability Y☐ N☐ **If no please detail why**

Comments on Assessment
